# Minimal Change in Cardiac Index With Increasing PEEP in Pediatric Acute Respiratory Distress Syndrome

**DOI:** 10.3389/fped.2019.00009

**Published:** 2019-01-29

**Authors:** Manpreet K. Virk, Justin C. Hotz, Wendy Wong, Robinder G. Khemani, Christopher J. L. Newth, Patrick A. Ross

**Affiliations:** ^1^Section of Critical Care, Department of Pediatrics, Texas Children's Hospital, Baylor College of Medicine, Houston, TX, United States; ^2^Department of Anesthesiology Critical Care Medicine, Children's Hospital Los Angeles, Keck School of Medicine of University of Southern California, Los Angeles, CA, United States; ^3^Critical Care Medicine, Valley Children's Hospital, Madera, CA, United States

**Keywords:** positive end-expiratory pressure, acute respiratory distress syndrome, mechanical ventilation, cardiac index, stroke volume

## Abstract

**Objective:** To determine if increasing positive end expiratory pressure (PEEP) leads to a change in cardiac index in children with Pediatric Acute Respiratory Distress Syndrome ranging from mild to severe.

**Design:** Prospective interventional study.

**Setting:** Multidisciplinary Pediatric Intensive Care Unit in a University teaching hospital.

**Patients:** Fifteen intubated children (5 females, 10 males) with a median age of 72 months (IQR 11, 132) and a median weight of 19.3 kg (IQR 7.5, 53.6) with a severity of Pediatric Acute Respiratory Distress Syndrome that ranged from mild to severe with a median lung injury score of 2.3 (IQR 2.0, 2.7).

**Measurements:** Cardiac index (CI) and stroke volume (SV) were measured on baseline ventilator settings and subsequently with a PEEP 4 cmH_2_O higher than baseline. Change in CI and SV from baseline values was evaluated using Wilcoxon signed rank test.

**Results:** A total of 19 paired measurements obtained. The median baseline PEEP was 8 cmH_2_O (IQR 8, 10) Range 6–14 cmH_2_O. There was no significant change in cardiac index or stroke volume with change in PEEP. Baseline median CI 4.4 L/min/m^2^ (IQR 3.4, 4.8) and PEEP 4 higher median CI of 4.3 L/min/m^2^ (IQR 3.6, 4.8), *p* = 0.65. Baseline median SV 26 ml (IQR 13, 44) and at PEEP 4 higher median SV 34 ml (IQR 12, 44) *p* = 0.63.

**Conclusion:** There is no significant change in cardiac index or stroke volume with increasing PEEP by 4 cmH_2_O in a population of children with mild to severe PARDS.

**Clinical Trial Registration:** The study is registered on Clinical trails.gov under the Identifier: NCT02354365.

## Introduction

Pediatric Acute Respiratory Distress Syndrome (PARDS) comprises a small fraction of critically ill children but continues to have high mortality up to 30–35% in severe PARDS patients (1–3). The use of positive end-expiratory pressure (PEEP) is an important component of mechanical ventilation strategies for PARDS to prevent the development of lung injury secondary to atelectasis (4, 5). A recent study has shown PEEP is often conservatively applied in PARDS, which may be associated with harm (6).

The reluctance to increase PEEP in PARDS is likely multifactorial, but frequent concerns relate to the effect of increased airway pressure on cardiac output. A variety of studies from the 1970s−1980s highlighted that PEEP can have deleterious effects on cardiac index and oxygen delivery, although this is generally only in a subset of hypovolemic patients, and this could be alleviated with judicious volume expansion (7–9).

Interestingly, there have been very few subsequent investigations of the impact of PEEP on cardiac output in children with ARDS, and recent recommendations by the Pediatric Acute Lung Injury Consensus Conference (PALICC) stress not only the importance of monitoring the cardiac output and oxygen delivery with PEEP titration for PARDS but that it is also a research priority to further evaluate the hemodynamic and potential barotrauma effects of higher PEEP titration (10, 11). It is possible given the recent findings delineating potential deleterious effects of PEEP (6) lower than ARDSNet protocol in PARDS along with the potential employment of transpulmonary pressure measurements to determine PEEP application in ARDS (12) PEEP levels higher than previously used will be applied in pediatric ARDS management.

Given these recommendations and insights, we sought to determine the hemodynamic effects of increasing PEEP in children with PARDS. We hypothesized that given the decreased compliance of the lungs in PARDS there would be little transmission of the increased airway pressure to the pulmonary vascular bed and thus minimal change in cardiac output.

## Materials and Methods

This is a prospective interventional study of mechanically ventilated children with lung injury admitted to a multi-disciplinary PICU. Inclusion criteria were as follows (13): intubated, mechanically ventilated, with two consecutive PaO_2_/FiO_2_ < 300 or SpO_2_/FiO_2_ < 265, aged <18 years. For inclusion, the patients needed to be receiving neuromuscular blockade, so that effects of respiratory system compliance could be adequately evaluated. However, neuromuscular blockade was not given specifically for the study. The severity of lung injury was classified using the Lung Injury Score (LIS) (14–16) or the Non-Invasive Lung Injury Score if an arterial blood gas was not available (17). The LIS is a composite score that includes pulmonary compliance, PEEP, Chest X-ray findings, and PaO_2_/FiO_2_ or SpO_2_/FiO_2_ ratios. A LIS > 2.5 is classified as severe PARDS (16). The study was approved by the Investigational Review Board at Children's Hospital Los Angeles and written, informed consent was obtained from the parents of all the participants in this study.

We measured Cardiac Index, stroke volume, ventilator settings, dynamic compliance, SpO_2_, and vital signs on the patient's clinician set baseline PEEP. We then increased the patient's PEEP by 4 cmH_2_O. We allowed at least 5 min for equilibration where pulse oximetry and tidal volumes measurements stabilized. We then re-measured Cardiac Index, stroke volume, ventilator settings, dynamic compliance, SpO_2_, and vital signs.

Cardiac index was measured non-invasively with an Ultrasound Cardiac Output Monitor (USCOM-1 A, USCOM Pty Ltd, Coffs Harbor, NSW, Australia), which uses a continuous wave Doppler to measure velocity of blood through the aortic valve. The product of velocity and the cross-sectional area of the aortic valve (obtained by patient age and weight) is stroke volume (SV) and SV multiplied by heart rate is CO. CI was calculated by dividing CO by patient body surface area. USCOM has previously been validated by our group against pulmonary artery catheter thermodilution cardiac output in pediatric patients (18). Oxygen delivery (DO_2_) was calculated using the equation, DO_2_ = CaO_2_ × CO. CaO_2_ was calculated using [1.39^*^hemoglobin^*^oxygen saturation (from pulse oximetry)]. We did not include the small dissolved component as PaO_2_ was not available on every patient. DO_2_ was indexed by the patient's body surface area.

### Statistical Analysis

Descriptive statistics were calculated. Changes in CI, SV and dynamic compliance at baseline PEEP and after PEEP 4 cmH_2_O higher were compared using Wilcoxon Signed Rank Test and Mann Whitney *U* test (Statistica Version 13, Statistica, Microsoft Corporation, Richmond, WA). A *p*-value < 0.05 was considered significant. Based on established norms, we sought to determine if the change in PEEP resulted in a clinically significant change in cardiac index, of ~15–20%. With a range of assumptions about the standard deviation of the cardiac index, we targeted a sample size of 15 patients. *Post-hoc* power analysis using the observed standard deviation demonstrated that we were adequately powered (0.8) to detect a 16.2% change in cardiac index.

## Results

Data from 15 children were included. The median age was 72 months (IQR 11, 132). The median measured weight was 19.3 kg (IQR 7.5, 53.6); median dynamic compliance for the measurements was 0.38 ml/cmH_2_O/kg (IQR 0.27, 0.50) [normal range 0.8–1.2 ml/cmH_2_O/kg]. Median lung injury score (LIS) was 2.3 (IQR 2.0, 2.7).

**Figure 1:**
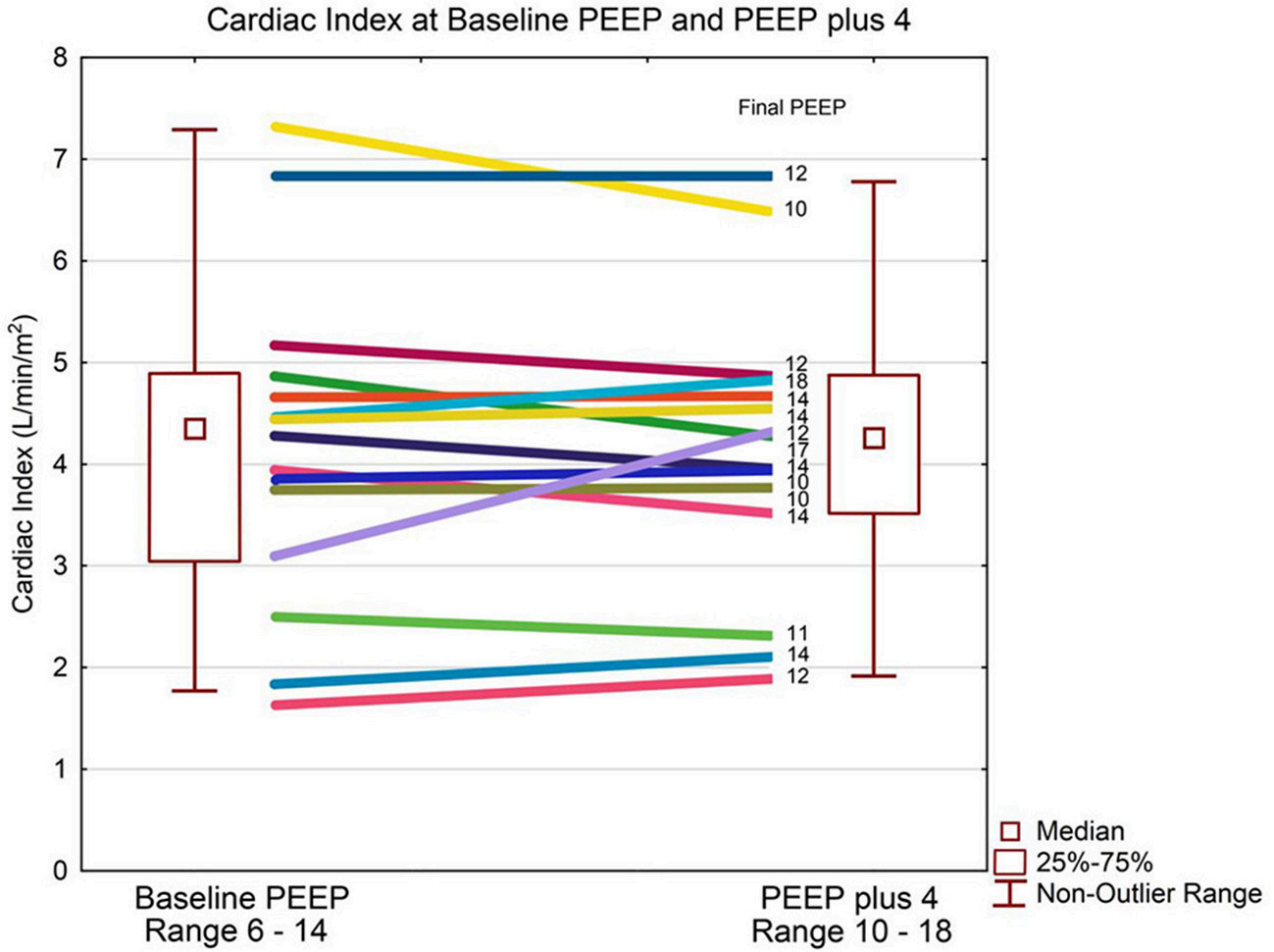
Cardiac index and PEEP. Data are presented as median, inter-quartile, and non-outlier range. The box plot represents the entire group and each patient's change in cardiac output is displayed as a line diagram with the change from baseline PEEP to PEEP plus 4 cmH_2_O. There is no statistically significant change in cardiac index at median PEEP of 8 cmH2O compared to median PEEP of 12 cmH2O.

**Figure 2:**
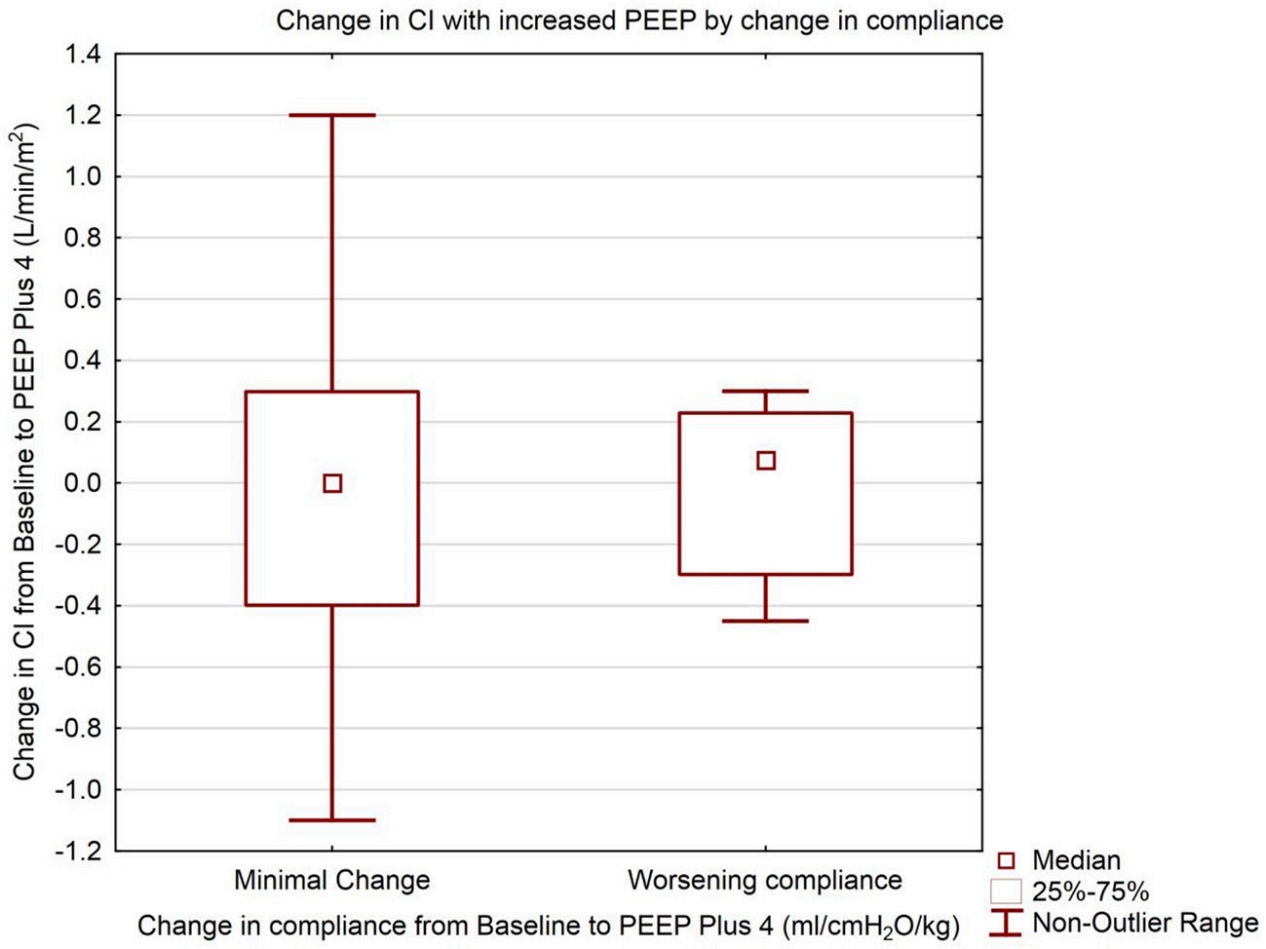
Change in Cardiac Index with increased PEEP. Groups separated by change in dynamic compliance of the respiratory system. Data are presented as median, inter-quartile, and non-outlier range. The left box represents change in cardiac index from Baseline to PEEP plus 4 cmH_2_O where there was minimal or no change in the respiratory compliance from Baseline to PEEP plus 4 cmH_2_O. The right box represents the change in cardiac index for subjects where the respiratory compliance worsened from Baseline to PEEP plus 4 cmH_2_O. There is no statistically significant difference between the change in cardiac index between the two groups (Mann Whitney *U* test, *p* = 0.91).

Patient demographics are shown in Table 1. The median baseline clinician set PEEP was 8 cmH_2_O (IQR 8, 10) Range 6–14 cmH_2_O. There was no statistically significant difference in median CI between baseline PEEP [4.4 L/min/m^2^ (IQR 3.4, 4.8)] and PEEP 4 cmH_2_O higher [4.3 L/min/m^2^ (IQR 3.6, 4.8)] (*p* = 0.65) as shown in Table 2 and Figure 1. There was no significant change in median indexed oxygen delivery (D'O_2_I) between baseline PEEP [493 ml/min/m^2^ (IQR 446, 687)] and PEEP 4 cmH_2_O higher [527 ml/min/m^2^ (IQR 471, 672)] (*p* = 0.98). Dynamic compliance decreased with the increase in PEEP for the overall population; baseline PEEP [0.38 ml/cmH_2_O/kg (IQR 0.27, 0.50)] and PEEP 4 cmH_2_O higher [0.31 ml/cmH_2_O/kg (IQR 0.22, 0.42)] (*p* = 0.0026). When looking at the individual patients, nearly all patients had no change in their CI between baseline PEEP and PEEP 4 cmH_2_O higher (see Figure 1). Additionally, there was no significant change in CI in subjects when stratifying by change in dynamic compliance as PEEP was increased to 4 cmH_2_O above baseline. The median change in CI with minimal change in compliance was 0.0 L/min/m^2^ (IQR −0.4, 0.3) the median change for those that had worsening compliance was 0.075 L/min/m^2^ (IQR −0.3, 0.23) (*p* = 0.91) (see Figure 2).

**Table 1:** Baseline demographics and characteristics.

**Patients demographics, baseline ventilator settings, vital signs**	**Median (IQR)**
	**No. (%)**
Age (months)	72 (11, 132)
Gender (female)	5/15 (33%)
Weight (kg)	19.3 (7.5, 53.6)
Baseline cardiac index (L/min/m^2^)	4.4 (3.4, 4.8)
Baseline stroke volume (ml)	26 (13, 44)
Baseline lung injury score	2.3 (2.0, 2.7)
**Baseline ventilator settings**	
Peak inspiratory pressure (cmH_2_O)	26 (24, 28)
Positive end-expiratory pressure (cmH_2_O)	8 (8, 10)
Mean airway pressure (cmH_2_O)	15 (14, 16)
FiO_2_	0.40 (0.38, 0.52)
Actual tidal volume (ml/kg)	5.9 (5.1, 7.3)
**Baseline vital signs**	
Heart rate (beats/min)	130 (109, 137)
Systolic blood pressure (mmHg)	100 (91, 108)
Diastolic blood pressure (mmHg)	54 (47, 64)
Mean arterial pressure (mmHg)	66 (62, 79)
Respiratory rate (bpm)	26 (24, 30)
Arterial oxygen tension (mmHg) (*n* = 13)	66 (59, 75)
Saturations %	96 (95, 97)
End-tidal CO_2_ (mmHg)	47 (39, 49)
Hemoglobin g/dL	10.2 (8.8, 11.3)
Measured Dynamic compliance (ml/cmH_2_O/kg)	0.38 (0.27, 0.50)
Normal Dynamic compliance (ml/cmH_2_O/kg)	0.8–1.2

*Data are presented as Median (interquartile range) or as Number (percentage)*.

**Table 2:** Changes in outcome measurements associated with increased PEEP.

	**Pre-intervention**	**Post-intervention**	***P*-value**
	**Median (IQR)**	**Median (IQR)**	
PEEP (cmH_2_O)	8 (8, 10)	12 (12, 14)	N/A
Cardiac index (L/min/m^2^)	4.4 (3.4, 4.8)	4.3 (3.6, 4.8)	0.65
Stroke volume (ml)	26 (13, 44)	34 (12, 44)	0.63
Dynamic compliance (ml/cmH_2_O/kg)	0.38 (0.27, 0.50)	0.31 (0.22, 0.42)	0.0026
Index oxygen delivery (ml O_2_/min/m^2^)	493 (446, 687)	527 (471, 672)	0.98
Heart rate	130 (109, 137)	131 (108, 141)	0.91

*Data are presented as Median (1st, 3rd interquartile range). Statistical differences between groups were evaluated with Wilcoxon Sign Rank Test*.

## Discussion

We found no statistically or clinically significant change in cardiac index, stroke volume or oxygen delivery with an increase in PEEP from baseline to a PEEP 4 cmH_2_O higher in infants and children with PARDS that ranged from mild to severe.

PEEP strategies are used in PARDS management to increase oxygenation and prevent alveolar collapse. The cardiopulmonary effects of higher PEEP during acute hypoxemic respiratory failure have been only rarely evaluated in the past in infants and children (7–9, 19) and deleterious effects were infrequent even on these patients who were clinically very sick. However, there still appears to be reluctance amongst pediatric clinicians to increase PEEP to higher levels (6, 20). The reluctance to increase PEEP amongst ARDS patients may be occurring for a variety of reasons including carry-over of mechanical ventilation practices and concern for cardiopulmonary interactions from management of post-operative cardiac surgery patients. A previous study in 15 children (21) with restrictive lung disease showed that PaO_2_ increased as PEEP was escalated from 0 to 15 cmH_2_O, probably by increasing functional residual capacity and decreasing intrapulmonary shunting. Interestingly, the increase in PaO_2_ was seen only at PEEP levels above 9 cmH_2_O. However, in these patients the higher PaO_2_ from increasing PEEP was counteracted with decreased pulmonary compliance (presumably indicating over distention) and lower cardiac output. As such, there was no net effect on oxygen delivery.

A study using impedance cardiography (22) as the measure of CO showed no significant change in CO as well as central venous oxygen saturations with incremental PEEP application from 0 to 15 cmH_2_O. This study was performed in normovolemic children with no pulmonary pathology. Their findings indicated that patients with adequate volume status with normally compliant lungs can likely compensate for the negative effects of PEEP on venous return. As is clear from the existing literature, the effect of increasing PEEP on lowering cardiac output is more pronounced when lungs are more compliant (8, 19, 23, 24). This suggests increasing PEEP with normally compliant lungs may cause over distension and transmission of the intrathoracic pressure to the heart resulting in decreased cardiac output. While this mechanism is well-understood, our previous investigation in children with mild to moderate decreases in lung compliance (some recovering from PARDS) noted a non-significant (<10%) reduction in cardiac output between PEEP levels of 0 and 12 cmH_2_O (19). Our current study showed a small decrease in compliance from 0.38 ml/cmH_2_O/kg (IQR 0.25, 0.50) at a median PEEP of 8 cmH_2_O to 0.31 ml/cmH_2_O/kg (IQR 0.20, 0.42) at higher PEEP with a median of 12 cmH_2_O. This was statistically significant (*p* = 0.0026) however unlikely to be clinically significant. There was no change in CI or oxygen delivery. In addition, there was no change in the vasoactive or ventilator support in any of the subjects as PEEP was escalated even though all of the subjects had final PEEP values of >10 cmH_2_O.

There are multiple potential benefits to the application of higher PEEP in PARDS. The cornerstone of ARDS therapy involves measures aimed at improving gas exchange while limiting injury to the pulmonary parenchyma. Animal studies (25) in normal anesthetized rats examined the effects of high positive inspiratory pressures without and with application of PEEP. In these studies, the animals with zero PEEP had alveolar edema, decreased dynamic compliance, severe hypoxemia and died within 1 h compared to the animals with PEEP of 10 cmH_2_O who had no alveolar edema and all survived. In addition to improved oxygenation and dynamic compliance when alveoli are under-recruited, PEEP can combat pulmonary edema from defective alveolar fluid clearance (26, 27) and cardiac dysfunction secondary to widespread vascular endothelial and epithelial injury in ARDS (28). Judicious use of PEEP will increase functional residual capacity (8, 21) decreasing left ventricular afterload and improving cardiac output and overall perfusion in the setting of multi-organ failure in ARDS (29). Recent data (6) from multicenter studies examining the effects of PEEP on PARDS have shown that PEEP lower than that recommended for the corresponding FiO_2_ per the ARDSNet protocol is an independent risk factor for higher mortality when compared with subjects on PEEP same as or higher than the ARDSNet recommendations. Finally, there is higher mortality with increasing dead space (V_D_/V_T_) in children with PARDS and PEEP can reduce V_D_/V_T_ if there is alveolar recruitment (30).

Our study has a number of limitations. The sample size is relatively small, although they did have a range of mild to severe lung injury with decreased respiratory system compliance. We limited PEEP increases to 4 cmH_2_O beyond baseline (clinically chosen) PEEP, so conclusions about larger PEEP changes cannot be made. While PEEP increases had no significant effect on cardiac index for the entire population, ultimately the effect of PEEP on cardiac function may differ from individual to individual. As such, it is certainly appropriate to consider that CI may fall in a subset of patients as PEEP is increased, but our data suggests this is a relatively uncommon occurrence. We did not specifically look at RV function and pulmonary hypertension, which could have an important impact on our reported cardiopulmonary interactions. Nonetheless, the median applied PEEP in our study was still higher than many physicians prescribe (19). The measurements of cardiac index were obtained using a non-invasive method rather than the accepted gold standard of thermodilution. However, this technique has been previously validated in children, and used by our group (18). The intention of our study was to evaluate the effect of PEEP on CI specifically, and was not specifically designed as a PEEP titration study evaluating other factors, such as lung volume, recruitment, oxygenation, dead space etc. Hence, conclusions are focused primarily on the effects on cardiac index. Finally, the measurements were not blinded to the baseline CI value prior to changing PEEP, introducing the possibility for bias.

In conclusion, in children who had a range of mild to severe PARDS, increases of PEEP of up to 4 cmH_2_O above the clinically chosen level resulted in no significant change in cardiac output or stroke volume. Judicious application of higher PEEP levels is unlikely to have significant adverse effects on cardiac output or oxygen delivery for most children with PARDS.

## Author Contributions

MV, JH, WW, RK, CN, and PR were involved in design of the study, recruitment of subjects, data collection, and manuscript preparation. All authors have approved the final draft for submission. Data analysis was done by PAR and has full access to the dataset.

### Conflict of Interest Statement

The authors declare that the research was conducted in the absence of any commercial or financial relationships that could be construed as a potential conflict of interest.

## Abbreviations

CaO_2_: arterial oxygen content
CO: cardiac output
CI: cardiac index
DO_2_: oxygen delivery
LIS: lung injury score
PARDS: pediatric acute respiratory distress syndrome.
